# A Rare Case of Persistent Lactic Acidosis in the ICU: Glycogenic Hepatopathy and Mauriac Syndrome

**DOI:** 10.1155/2016/6072909

**Published:** 2016-07-25

**Authors:** Kirsten S. Deemer, George F. Alvarez

**Affiliations:** ^1^Department of Critical Care Medicine, South Health Campus ICU, 4448 Front Street SE, Calgary, AB, Canada T3M 1M4; ^2^Department of Critical Care Medicine, University of Calgary, AB, Canada

## Abstract

Mauriac syndrome is a rare disorder that can present with the single feature of glycogenic hepatopathy in children and adults with poorly controlled diabetes mellitus. An often underrecognized finding of glycogenic hepatopathy is lactic acidosis and hyperlactatemia. Primary treatment of glycogenic hepatopathy is improved long-term blood glucose control. Resolution of symptoms and hepatomegaly will occur with improvement in hemoglobin A1C. We present here a case of a young adult female presenting to the intensive care unit with Mauriac syndrome. This case demonstrates* exacerbation* of lactic acidosis in a patient with glycogenic hepatopathy treated for diabetic ketoacidosis with high dose insulin and dextrose.

## 1. Introduction

Lactic acidosis is a common finding in critically ill patients admitted to the intensive care unit (ICU) and it is associated with increased mortality [1, 2]. An anion gap and pH of less than 7.35 are not required for a definition of lactic acidosis as additional causes for anion gap and metabolic alkalosis often exist [3]. Seventy percent of lactate metabolism to glucose takes place in the liver via gluconeogenesis. Anaerobic glycolysis generates pyruvate, NADA, and H+, which is converted into lactate. When lactate production rises and exceeds that of consumption, hyperlactatemia and lactic acidosis result [3].

The most common cause of lactic acidosis in the ICU is type A. Currently there is debate among researchers surrounding the pathogenesis of some forms of type A lactic acidosis and whether it is attributed to tissue hypoxia and anaerobic glycolysis or simply an adrenergic response during stress and increased aerobic glycolysis [4]. Nevertheless, it is often found in disease states such as cardiogenic and hypovolemic shock, sepsis, trauma, and severe hypoxemia [1, 3].

Type B lactic acidosis is less commonly seen in critically ill patients and occurs without evidence of tissue hypoperfusion or shock [3, 5]. Several etiologies of type B lactic acidosis have been described, such as drug metabolites, toxins, congenital enzyme deficiencies, grand Mal seizures, liver failure, hematologic malignancies, renal disease, ethanol intoxication, thiamine deficiency, and diabetes mellitus (DM) [1–3, 5].

The correlation between DM and lactic acidosis is previously described in diabetic ketoacidosis (DKA); however, it can also be seen in clinically well patients with diabetes and glycogenic hepatopathy [6]. This paper will describe a case of persistent lactic acidosis in a young adult female with poorly controlled diabetes and hepatomegaly.

## 2. Case Presentation

An 18-year-old female with a history of type 1 DM diagnosed at the age of 6 presented to the ER with complaints of nausea, vomiting, and diarrhea. Her blood glucose was 22 mmol/L with an anion gap of 25 and lactate of 2.1 mmol/L. Urine tested positive for ketones. She complained of three days of upper respiratory tract infection symptoms and stated that she had high blood sugars for days but did not take enough insulin and omitted a dose with her last meal. She had two prior admissions to hospital with DKA and was noted to have chronically suboptimal glucose control (hemoglobin A1C 11.3%). The patient was afebrile and blood and urine cultures were negative for bacteria.

After one day in hospital with insulin and dextrose infusions, the anion gap closed and the patient was converted to subcutaneous insulin. However, the following day, plasma lactate levels were noted to be 3.9 mmol/L with an elevated anion gap. Insulin and dextrose infusions were restarted due to concerns of recurrent DKA. Due to persistent lactic acidosis and labor-intensive DKA management, the patient was moved to the ICU.

Upon examination, the patient looked well. She was hemodynamically stable and afebrile and had no respiratory distress. She had no signs or symptoms of shock. Abdomen on palpation was soft, nondistended, and nontender. Liver was palpated at 8 cm below the costal margin with no palpable splenomegaly. Blood glucose was 5.8 mmol/L with an insulin infusion at 3 units/hour and 5% dextrose infusion at 100 mL/hour. Serum total bilirubin was 2 umol/L, ALT 36 U/L, alkaline phosphate was 120 U/L, and lipase was 12 U/L. Abdominal ultrasound revealed a liver span of 27 cm with hepatosteatosis and mild splenomegaly.

**Figure 1:**
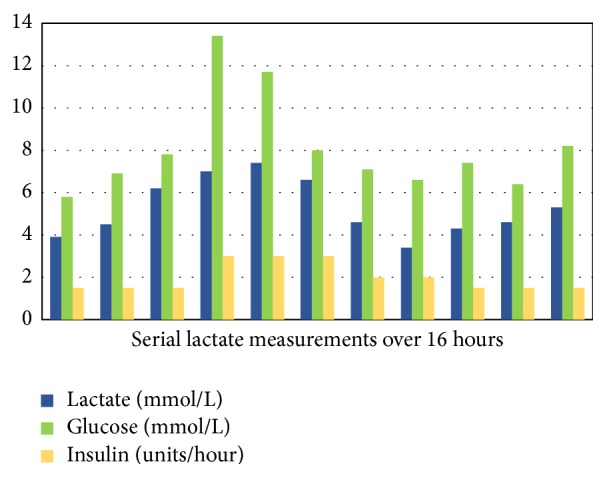
Persistent lactic acidosis with dextrose and insulin therapy in an 18-year-old female patient with hepatomegaly.

Serial measurements of arterial blood gases revealed persistent lactic acidosis and anion gap despite insulin and dextrose infusions (Figure 1).

The patient was transitioned from insulin infusion to subcutaneous insulin the following day. Her blood sugars stabilized in the range of 6–9 mmol/L and her last measured lactate was 5.3 mmol/L. She was discharged home with an endocrinologist referral for a suspected diagnosis of glycogenic hepatopathy and Mauriac syndrome.

**Figure 2:**
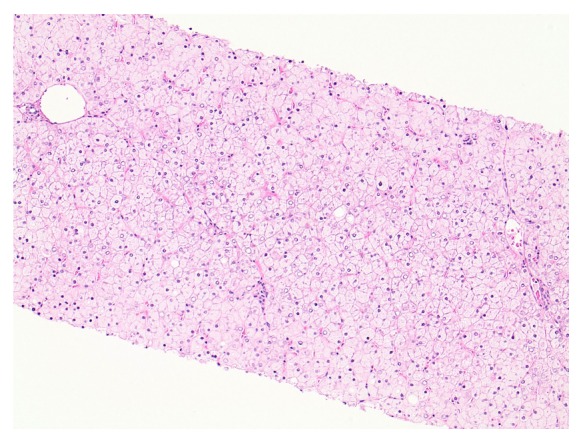
Liver Biopsy. Diffusely pale and swollen hepatocytes with numerous glycogenated nuclei. There is no architectural distortion, significant inflammation, or large droplet steatosis.

**Figure 3:**
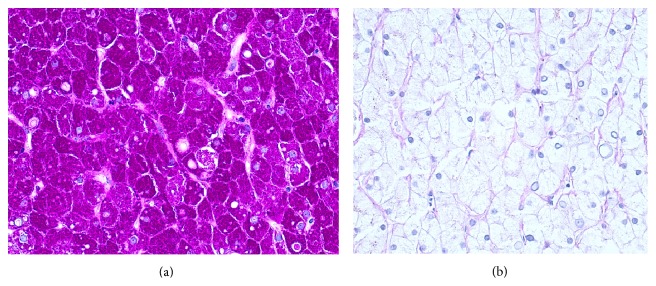
(a) Liver Biopsy. Periodic Acid-Schiff stain-positive for glycogen accumulation. (b) Glycogen abolishes after pretreatment with diastase.

Subsequently, the patient underwent a chronic liver disease screen. Alpha-1 antitrypsin, ceruloplasmin, iron studies, viral hepatitis, celiac and immunological testing were normal. A FibroScan showed no evidence of hepatic fibrosis. After discharge, an abdominal ultrasound again revealed hepatomegaly with liver span of 24 cm. Mitochondrial disorder and respiratory chain defects were ruled out. A liver biopsy revealed numerous hepatocytes with glycogenated nuclei, abundant cytoplasmic and nuclear glycogen deposits, and no large droplet steatosis (Figure 2). Periodic Acid-Schiff (PAS) stain was positive for glycogen accumulation (Figure 3(a)), which was abolished with diastase pretreatment (Figure 3(b)). Therefore, a diagnosis of Mauriac syndrome was given with the only manifestation being that of glycogenic hepatopathy with lactic acidosis. The patient was referred to a diabetes management clinic for further optimization of blood glucose. Lactate level six months following discharge was 4.9 mmol/L.

## 3. Discussion

First described in 1930, Mauriac syndrome is typically diagnosed in young patients with poorly controlled type 1 DM with growth retardation, delayed puberty, cushingoid features, hypercholesterolemia, and hepatomegaly [7, 8]. However, the single presenting feature of Mauriac syndrome can be glycogenic hepatopathy in both adults and children [6].

Glycogenic hepatopathy is an underrecognized and uncommon complication of poorly controlled DM type 1 manifested by hepatomegaly, abdominal pain, nausea and vomiting, elevated serum transaminases, and elevated plasma lactate levels [9, 10].

### 3.1. Pathophysiology of Glycogenic Hepatopathy

Glucose is transported into hepatocytes without the aid of insulin. In Mauriac syndrome, hepatic glycogen deposition is achieved during hyperglycemia. Large amounts of insulin drive glycogen synthesis and decrease gluconeogenesis and glycogenolysis. Further insulin administration and hyperglycemia facilitate further glycogen synthesis, which creates congested hepatocytes resulting in storage overload [10, 11].

Subsequent episodes of DKA and hyperglycemia only compound the problem. Inherent in the treatment of DKA are high amounts of insulin and dextrose, which promotes glycogen synthesis within hepatocytes. The resultant manifestation is hepatomegaly and sometimes elevated transaminases but preserved synthetic liver function in patients with poor glucose control and a history of DKA admissions [8, 10].

A poorly recognized consequence of glycogenic hepatopathy is lactic acidosis. Fitzpatrick et al. described half of all pediatric study participants with hepatopathy of Mauriac syndrome having elevated lactate levels despite no signs of illness or DKA [7]. Elevation of lactate in chronic liver diseases, such as cirrhosis, may be partially due to accelerated glycolysis in the splanchnic region [12]. However, the mechanism of hyperlactatemia in Mauriac syndrome and type B lactic acidosis is poorly understood [5]. A reduction in gluconeogenesis in the liver may raise lactate levels in the body. Therefore, lactic acidosis in Mauriac syndrome could be explained by reduced gluconeogenesis and lack of conversion of pyruvate to glucose [12].

Given that large amounts of insulin and glucose are required for the development of glycogenic hepatopathy, this would explain the persistent and worsening lactic acidosis in our patient with typical DKA treatment of dextrose and insulin infusions [10]. In patients with poorly controlled diabetes, initial treatment of hyperglycemia with insulin has been shown to cause transient elevation of liver enzymes [13].

### 3.2. Diagnosis and Treatment

Diagnosis of glycogenic hepatopathy involves ruling out infectious disease, oncologic, autoimmune, metabolic (glycogen storage disease) or, more commonly in diabetic patients, nonalcoholic fatty liver disease [10]. Imaging includes abdominal ultrasonography; however, ultrasound does not differentiate fatty liver from glycogen overload [8, 11, 13]. Liver biopsy is the gold standard in diagnosing glycogenic hepatopathy and reveals marked glycogen accumulation within hepatocytes leading to enlarged, pale cells [8, 10]. Mild large droplet steatosis may be present [9].

Treatment of Mauriac syndrome and glycogenic hepatopathy involves improved blood glucose management [11, 13]. Resolution of symptoms, normalizing of liver enzymes and resolved hepatomegaly, has been demonstrated with only minor improvements to hemoglobin A1C levels [11].

## 4. Conclusion

Mauriac syndrome is a rare complication of poorly controlled DM and may present with lactic acidosis. This case demonstrates lactic acidosis exacerbated by high dose insulin and dextrose therapy. Further research is required to explain the pathophysiology of lactic acidosis in glycogenic hepatopathy.
